# Immune Modulation by Design: Using Topography to Control Human Monocyte Attachment and Macrophage Differentiation

**DOI:** 10.1002/advs.201903392

**Published:** 2020-04-28

**Authors:** Matthew J. Vassey, Grazziela P. Figueredo, David J. Scurr, Aliaksei S. Vasilevich, Steven Vermeulen, Aurélie Carlier, Jeni Luckett, Nick R. M. Beijer, Paul Williams, David A. Winkler, Jan de Boer, Amir M. Ghaemmaghami, Morgan R. Alexander

**Affiliations:** ^1^ School of Life Sciences University of Nottingham Nottingham NG7 2RD UK; ^2^ School of Computer Science University of Nottingham Nottingham NG8 1BB UK; ^3^ School of Pharmacy University of Nottingham Nottingham NG7 2RD UK; ^4^ Department of Biomedical Engineering and Institute for Complex Molecular Systems Eindhoven University of Technology 5600 EB Eindhoven The Netherlands; ^5^ Department of Cell Biology Inspired Tissue Engineering MERLN Institute for Technology‐Inspired Regenerative Medicine Maastricht University 6229 ET Maastricht The Netherlands; ^6^ University of Nottingham Biosdiscovery Institute and School of Medicine University of Nottingham Nottingham NG7 2UH UK; ^7^ University of Nottingham Biodiscovery Institute and School of Life Sciences University of Nottingham Nottingham NG7 2RD UK; ^8^ La Trobe Institute for Molecular Science La Trobe University Bundoora 3042 Australia; ^9^ School of Pharmacy University of Nottingham Nottingham NG7 2RD UK; ^10^ Monash Institute of Pharmaceutical Sciences Monash University Parkville 3052 Australia; ^11^ CSIRO Data61 Parkville 4069 Australia

**Keywords:** biomaterials, high‐throughput screening, immune‐modulation, topography

## Abstract

Macrophages play a central role in orchestrating immune responses to foreign materials, which are often responsible for the failure of implanted medical devices. Material topography is known to influence macrophage attachment and phenotype, providing opportunities for the rational design of “immune‐instructive” topographies to modulate macrophage function and thus foreign body responses to biomaterials. However, no generalizable understanding of the inter‐relationship between topography and cell response exists. A high throughput screening approach is therefore utilized to investigate the relationship between topography and human monocyte–derived macrophage attachment and phenotype, using a diverse library of 2176 micropatterns generated by an algorithm. This reveals that micropillars 5–10 µm in diameter play a dominant role in driving macrophage attachment compared to the many other topographies screened, an observation that aligns with studies of the interaction of macrophages with particles. Combining the pillar size with the micropillar density is found to be key in modulation of cell phenotype from pro to anti‐inflammatory states. Machine learning is used to successfully build a model that correlates cell attachment and phenotype with a selection of descriptors, illustrating that materials can potentially be designed to modulate inflammatory responses for future applications in the fight against foreign body rejection of medical devices.

## Graphical abstract

Using an unbiased screen of an algorithm generated topographical library in combination with machine learning, this study identifies topographies which instruct both the attachment and polarization of human macrophages in the absence of exogenous cytokines.

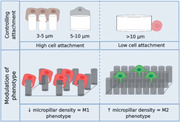

Excessive inflammation, driven by adverse immune responses, is a major impediment to the long‐term success of many medical devices which becomes more evident as we live for longer.^[^ ^1^, ^2^, ^3^ ^]^ There remains a pressing need for better “immune‐instructive” materials that could leverage the immune system's pro‐healing potential to promote better integration or tolerance of medical devices. Research aiming to discover novel biomaterials has grown substantially in recent years, using bioinspired or high throughput screening strategies to identify material cues to instruct desirable cellular phenotypes.^[^ ^3^, ^4^, ^5^, ^6^, ^7^, ^8^ ^]^ Immune modulation using micro topography provides significant opportunity to contribute to control of the host‐biomaterial interface.^[^ ^9^ ^]^ With limited understanding of the cellular and molecular mechanisms in the complex crosstalk between immune cells and materials, an unbiased screening approach becomes an attractive way to discover new and desirable biomaterial functionality.^[^ ^7^, ^9^, ^10^ ^]^

One of the key components in this complex process is the initial inflammatory response mediated by macrophages that play a central role in orchestrating foreign body responses which lead to acceptance/rejection of the material in the human body.^[^ ^11^ ^]^

Macrophages are the sentinels and regulators of the immune system that are present in nearly all tissues of the body and as such, encounter a variety of environments and stimuli, both chemical and physical.^[^ ^12^, ^13^, ^14^ ^]^ The high plasticity in macrophage phenotype and their ability to efficiently respond to micro environmental cues provide opportunities for development of “immune‐instructive” materials.^[^ ^15^ ^]^ The ability to modify or modulate the polarization status of macrophages using materials is emerging as an approach to tackle inflammatory diseases and as a therapeutic opportunity if we know how to design biomaterials design to achieve the desired responses.^[^ ^10^, ^16^, ^17^, ^18^ ^]^ Current strategies to modulate these immune cell responses include modification of the surface chemistry or incorporation of bioactive components.^[^ ^19^ ^]^ The focus of these modifications being to reduce the amount and identity of protein adsorption, modulated by different surface chemistries, and change the initial cell attachment to prevent, reduce, or modify cell interaction and inflammatory processes occurring at the material interface.^[^ ^11^, ^20^, ^21^ ^]^ However, previous studies investigating materials design approaches to surface topographies (in the micron range), have provided data on cell attachment in the context of modulating cell shape and morphology as the underpinning mechanism driving of polarization changes.^[^ ^22^, ^23^, ^24^ ^]^ Modification of the surface topography of biomaterials at the micron level has previously been shown to be relevant to improving outcome measures such as fibrotic encapsulation.^[^ ^25^ ^]^ Random roughened surfaces, introduction of channels, and micro pillars and pits have been used to achieve changes in macrophage adhesion, spreading, cytoskeletal remodeling, and transcriptomic profiles, all of which have significant implications for the clinical outcome of a biomaterial.^[^ ^17^, ^18^, ^22^, ^26^, ^27^ ^]^ It is increasingly evident that the ability to modulate macrophages is inherently linked to their physical interaction with the local microenvironment with a significant impact on the downstream gene expression and phenotypic response.^[^ ^17^, ^18^ ^]^ Given the limited understanding of the interplay between macrophages and the physical cues of their environment, we chose to pursue an unbiased, high throughput screening approach using the TopoChip platform. We aim to discover topographies that provide a greater cell‐instructive drive than the simple geometries already investigated, and to generate sufficient data with which to investigate this with the aspiration of identifying generalizable findings of material structure‐cell response relationships.^[^ ^28^ ^]^ We employed the TopoChip technology platform which allows 2176 unique, mathematically defined surface topographies to be screened on each chip in duplicate for their influence on cell response in a high throughput manner.^[^ ^29^, ^30^, ^31^, ^32^, ^33^ ^]^ Using algorithms to build topographical features from primitive features (circle, triangle, and rectangle; sized 3–23 µm in diameter and 10 µm in height), 2176 designs were arranged periodically to form 290 × 290 µm TopoUnits. Here, we present the first report of an unbiased screen to investigate how this diverse library of topographies influences human macrophage attachment and phenotype.

Monocytes were isolated from peripheral blood mononuclear cells (PBMCs) from anonymous human blood samples obtained in the form of buffy coats, using CD14 magnetic beads (Miltenyi Biotec) and used for the TopoChip screening (**Figure** **1**a). These cells were at no point stimulated or exposed to any exogenous cytokines. Using oxygen plasma etched polystyrene TopoChips in a serum containing medium, high throughput screening was carried out using monocytes obtained from five independent donors. Rank order analysis of the cell attachment (Figure 1b) was compared across the different donors showing consistency for the high and low attachment surfaces (Figure S1, Supporting Information) indicating the attachment measured was statistically robust. The flat, planar surface had a mean attachment of six cells per TopoUnit (indicated by blue dotted line; Figure 1b). Overall, monocyte attachment was significantly higher in the presence of topographical features compared to the flat planar control surface. Amongst the patterned surfaces, there was clear differential attachment of monocytes to specific surface types ranging from over 100 cells (per TopoUnit area) on high attachment TopoUnits compared to <10 for low attachment topographies (Figure 1c).

**Figure 1:**
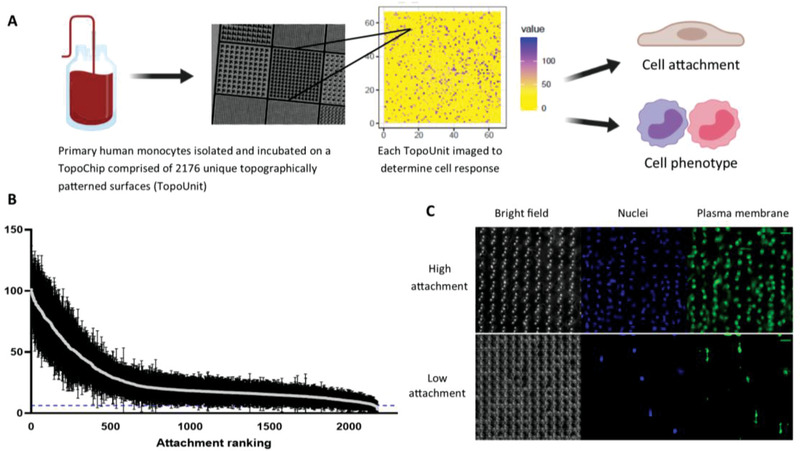
High throughput screening of monocyte attachment to topographically patterned surfaces. a) CD14+ human monocytes were isolated and cultured on polystyrene TopoChips for 3 days in the absence of any exogenous cytokines. Each data point represents the mean ± standard deviation from nine TopoChips tested across five independent donors; dotted line indicates flat planar surface Each TopoUnit was imaged independently analyzed using CellProfiler to determine cell attachment the flat, planar surface had a mean attachment of six cells per TopoUnit indicated by blue dotted line. b) Attachment performance rank order of mean monocyte attachment (per individual TopoUnit) was calculated to compare TopoUnit performance. c) Cells were stained with a plasma membrane dye and counterstained with DAPI to quantify attachment. Representative images of high and low attachment TopoUnits (from five independent donors; scale bar = 20 µm).

In order to understand the role of specific surface features and characterize the differential attachment we employed a computational regression analysis. The dataset was pre‐processed and aggregated using the mean cell attachment across all donors and TopoUnits with removal of data with a signal to noise ratio (SNR) < 2. Subsequently, multiple regression modeling using Gradient Boosting Regression was applied to the data to correlate cellular attachment with a library of 65 specific surface feature descriptors (Table 1, Supporting Information). These were created from a combination of parameter values used to construct the features and parameters generated from image analysis (bright field images) that describe characteristics of surface feature area and shape. The model generated an *R* ^2^ of 0.9 and 0.75 for the macrophage attachment training and test sets respectively (Figures S2b and 2c, Supporting Information) suggesting the models adequately describe the dataset. Descriptors that highlighted the size of the individual components of the TopoUnits were dominant in the model, specifically the presence of micro pillars with a small surface pattern area (Pattern Area), capturing quantitatively the differences between high and low attachment TopoUnits seen in Figure 1 topographies. These models are shown to accurately predict the responses to test sets containing combinations of known and novel topographies and show that these surface features have the largest impact on the biological properties. This indicates the potential for using high throughput data sets generated from TopoChip screening to train robust surface structure‐cell response models using machine learning.

In order to identify the specific physical feature responsible for macrophage attachment, surface feature importance was calculated and expressed as Shapley Additive exPlanantion (SHAP) values to determine the most importance surface parameter for macrophage attachment. The performance of this model and the descriptors that contributed most strongly to cell attachment depend most strongly on the presence of cylindrical micro‐pillars in the TopoUnits and a number of associated structural descriptors (see Figure S2b, Supporting Information). To understand the role of micro‐pillar size specifically, we clustered the cellular attachment data using k‐means, resulting in three clusters with high (5.5%), medium (20%), and low macrophage attachment (74.5%) and correlated those groups to TopoUnit performance. The highest attachment of macrophages across the TopoChip was noted on the surfaces that contained micropillars 5 micron in diameter (based on the mean Pattern Area) (**Figure** **2**a). Increased attachment was also noted on TopoUnits with micropillars up to 10 micron, however, this was the critical size above which macrophage attachment diminished significantly (Figure S3, Supporting Information). Confocal imaging of macrophages on high and low attachment TopoUnits indicated specific cell‐surface interactions with respect to feature size and cell attachment. On low cell attachment TopoUnits (with surface features > 10 µm) the cell adhesion occurred in between the large features (Figure 2b) in contrast to high attachment surfaces where micropillars appeared to be completely engulfed by the macrophages (Figure 2d). This ability of topography to modulate macrophage attachment was also unchanged in the presence of pre‐adsorbed extracellular matrix (ECM) components (fibronectin or collagen‐I; Figure S4, Supporting Information) in contrast to the flat planar area where we observe a characteristic increase in cell binding to an ECM coated surface, suggesting that the elevated cell attachment induced by topography exceeds the driving force of ECM pre‐adsorption. The observation of engulfment of micropillars as the dominant differentiator of attachment in this library is in line with previous observations of macrophage interaction with surfaces and microparticles in this size range.^[^ ^26^ ^]^ It would be interesting to vary the height of the topographical patterns, currently fixed at 10 µm, however our fabrication technique is limited to a single height for each chip. Future work will explore different heights generated using additive manufacturing where more design freedom is inherently afforded by the process.

**Figure 2:**
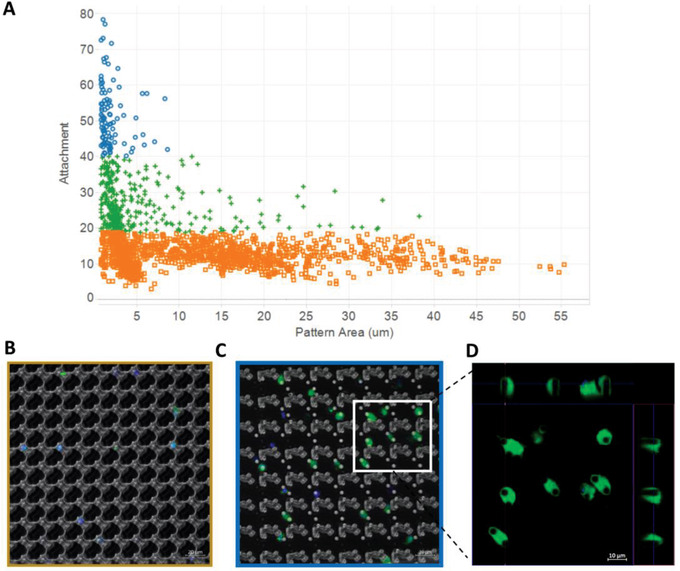
Macrophage attachment is mediated by small circular pillars. A) Macrophage attachment versus total pattern area (µm^2^) with the size of topographical features categorized as high (blue), medium (green), or low (orange) attachment. Categories of macrophage attachment were determined by cluster analysis using Euclidian distance. Representative composite confocal images of B) low attachment and C) high attachment TopoUnits with inset D) orthogonal views of Z‐stack images of macrophage plasma membrane (green) indicates cellular engulfment of the entire cylindrical pillar feature (also counterstained with DAPI (blue); Scale bar = 10 µm).

In order to determine if the adsorption of biomolecules was different on different TopoUnits, we characterized the surface of the topographies using in situ mass spectrometry before and after media exposure using a time of flight secondary ion mass spectrometry (ToF SIMS). A selection of high and low attachment TopoUnits were incubated with RPMI media (with 10% fetal bovine serum) for 1 h or left untreated and subsequently analyzed using the 3D SIMS instrument specifically for 2D surface chemical imaging (see Experimental Section).^[^ ^34^ ^]^

Assessment of the medium treated and un‐treated TopoUnits 3D SIMS data (Figure S2a, Supporting Information) revealed differences, primarily associated with protein adsorption on the medium treated TopoUnits. This is illustrated using the secondary ion peaks *m*/*z* 84 (C_5_H_10_N^+^) and 91 (C_7_H_7_^+^) representing protein (a generic lysine fragment) and the polystyrene base chemistry respectively (Figure S5a,b, Supporting Information).^[^ ^35^, ^36^ ^]^ A significant decrease in polystyrene signal following media incubation and an associated increase in protein coverage of the surface chemistry was observed, illustrating the coverage of the substrate with proteins (Figure S5c,d, Supporting Information). Comparison of these secondary ion intensities on representative high and low attachment surfaces was used to determine if differential biomolecule adsorption to TopoUnits was a factor in the cell response. Secondary ion peak intensities of post‐medium incubation topographies indicated no significant difference between high and low attachment surfaces. While SIMS is limited to fingerprint identification of proteins, comparison of the post incubation spectra suggests that no large compositional differences are observed between the different TopoUnits (Figure S6, Supporting Information). The total protein amount was quantified using X‐ray photoelectron spectroscopic (XPS) analysis indicating that there is a strongly adsorbed protein layer of ca. 1 nm thick (dehydrated) which equates to approximately 30% coverage and was not correlated with macrophage cell attachment (Figure S7, Supporting Information). To probe the ion coverage and distribution of the selected topographies we used high resolution imaging (utilizing delayed extraction of the mass analyzer) which showed a uniform surface distribution of the peak *m*/*z* 42 (CNO^−^) (unspecific protein marker) in the medium incubated samples (Figure S8, Supporting Information). Protein deposition was observed across both high and low attachment surfaces, however, there was no discernible difference in the spatial distribution in terms of the apical, lateral, or basal surface of the TopoUnit surfaces. Using the complementary high spatial resolution and chemical characterization of the surface chemistry provides confidence in assigning the cell response driver to the topography rather than changes in surface chemistry for these micro patterned surfaces.

After screening a range of topographies for monocyte attachment and gaining insight into the structure–function relationship, we investigated the influence of surface topography on macrophage phenotype. Macrophage polarization is a key determinant in maintaining tissue homeostasis after injury and is known to correlate with clinical outcome of implanted medical devices. Using a high throughput approach, the measurement and characterization of defining features of polarization status is a trade‐off between a large number of topographies (screening) and detailed cellular phenotyping. Polarization of naïve (M0) macrophages to pro‐ (M1) or anti‐inflammatory (M2) phenotypes.

Harnessing macrophage polarity presents a unique opportunity to control inflammation, prevent rejection, and accelerate integration of biomaterials and medical devices. We hypothesized that the surface topography would play a key role in this biological process.

In order to investigate this, monocytes were incubated on TopoChips in the absence of exogenous cytokine stimulation for 6 days prior to phenotypic characterization. Macrophage phenotypic status was determined using cell surface markers known to be associated with M1/M2 phenotypes (calprotectin and mannose receptor for M1 and M2, respectively).^[^ ^20^ ^]^ In order to determine phenotypic responses, the M2/M1 ratio was calculated (per cell) and normalized to the flat, planar TopoUnit on each chip respectively. Those TopoUnits with a SNR of <2 were removed from further analysis.

Overall, the proportion of the three potential phenotypes (M2/M0/M1) across the TopoChip indicated there was a range of phenotypic responses to different topographies, and no one predominant macrophage polarization state (**Figure** **3**a). Furthermore, cluster analysis identified a relationship between cell number per TopoUnit and M2 polarization status (Figure 3b). Modulation of macrophage phenotype was reflected by clear changes in surface marker ratios ranging from 1.82 to 0.8 in Figure 3c–e (flat planar surface = 1.2). In comparison, cytokine polarized controls where we observed a range from 2.13 to 0.41 for M2–M1, respectively (Figure S12, Supporting Information).

**Figure 3:**
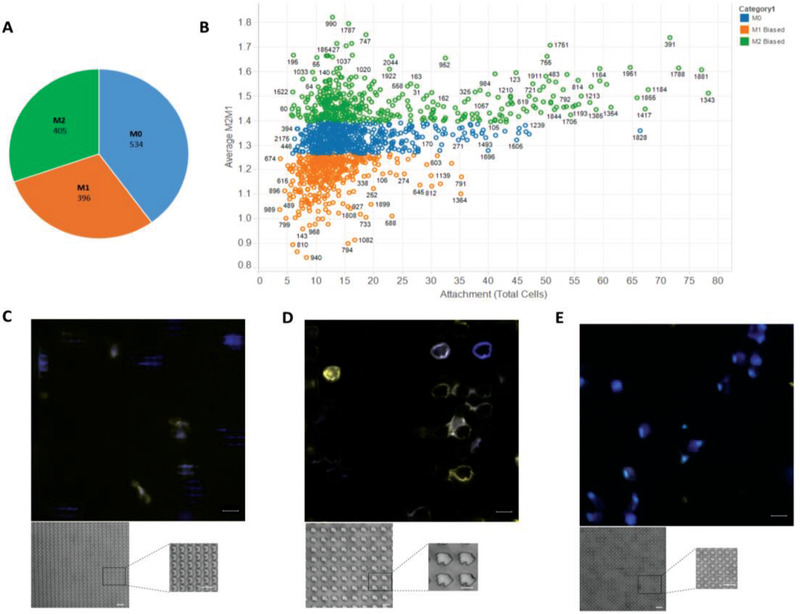
Phenotypic screening of monocyte attachment to topographically patterned surfaces. CD14+ human monocytes were isolated and cultured on plasma treated polystyrene TopoChips for 6 days in the absence of any exogenous cytokines. Each TopoUnit was imaged and independently analyzed using CellProfiler to determine phenotype based on mean fluorescence intensity of calprotectin (M1) and mannose receptor (M2) per cell. A) Circle chart representing the relative proportions of the macrophage phenotypic response (with SNR>2) from the TopoChip B) Scatter plot of TopoUnit phenotype (average M2/M1 ratio) and macrophage attachment. Categories determined by hierarchical cluster analysis using Euclidian distance (phenotype represented in blue—M0, orange—M1 biased and green —M2 biased). C–E) Representative fluorescent images of C) M0, D) M1, and E) M2 biased TopoUnits with insets of higher magnification bright field images of the topographical features. Calprotectin (M1) expression represented in yellow and mannose receptor (M2) in blue. Scale bar = 20 µm.

Similarly, for macrophage attachment, we developed a model to describe the macrophage phenotype relative to the surface parameter descriptors to provide information on relevant physical surface structure descriptors. As cell attachment and polarization were both important factors, we trained machine learning models to predict a composite dependent variable that incorporated both phenotype and attachment: log(M2/M1) x cell attachment. This variable has large positive or negative values to enable identification of TopoUnits with high attachment and a specific phenotype (M2 or M1) and low values for those with low attachment/phenotype. Therefore, the units of most interest exhibit either the most positive value for the composite variable to the anti‐inflammatory phenotype class (M2), or the materials with most negative values for the composite variable into the pro‐inflammatory class (M1). The anti‐ and pro‐inflammatory groups were defined after clustering the dataset and selecting those instances from the clusters with the highest and lowest values found for the composite variable.

The regression model for polarization generated an *R* ^2^ of 0.84 and 0.56 for the macrophage phenotype training and test sets respectively, and SHAP values indicated key topographical parameters that drive macrophage phenotype modulation (Figure S10b,c, Supporting Information). Specifically, the features associated with phenotypic changes related to feature size described by Pattern Area and most dominantly Pattern Area_min, the smallest in the TopoUnit. The spacing between features (described using MaxInscribedCircles) as a function of micropillar density was also an important driver of phenotype. Further correlation analysis of the top 50 M2 or M1 TopoUnits showed that these features were all statistically significant in their ability to modulate a specific macrophage phenotype (Figure S11a–d, Supporting Information). Interestingly, it is the combination of these key surface structures that is responsible for driving changes in macrophage phenotype and provides a design rule for physical surface design. This is reflected in the representative (inset) bright field microscopy images of TopoUnits in Figure 3c–e whereby M1 phenotype is driven by larger, more disperse surface features compared to smaller, denser micro‐pillars driving an M2 phenotype. Further testing on a range of selected topographies validated this observation with corresponding responses seen in TNF*α* and IL‐10 intracellular staining (Figure S12, Supporting Information). Our findings expand on previous work by Bartneck et al. whose results concluded that macrophages cultured on small cylindrical posts (20 µm diameter) showed a high M2 and low M1 surface marker profile, compared to cells on micro patterned grooves which showed increased expression of surface markers characteristic of M1 (pro‐inflammatory) status.^[^ ^26^ ^]^ However, by screening thousands of topographies to sample a wide design space and using hundreds of descriptors, we have been able to determine the importance of micropillars compared to a wide range of other shapes and identify the importance of their size and density in the control macrophage behavior. The observations noted here are in line with studies focused on the dependence of macrophage phagocytosis on shape and size of microparticles in 3D. Champion et al. reported that shape, more specifically, the localized shape at the point of initial contact determines whether macrophages initiate phagocytosis or simply spread on particles.^[^ ^37^, ^38^, ^39^ ^]^ This may also explain the propensity for the cells to preferentially interact with a topography compared to flat planar surfaces.

Given the increasing understanding and importance of physical shape and cues from topographical patterned surfaces, the translation from in vitro to in vivo validation is a key step towards clinical application. As a proof of concept, we tested the structural stability of high attachment micro topographical features in an animal model and show evidence of cell attachment to the TopoUnit surface (Figure S13, Supporting Information). Future work will explore and characterize biological mechanisms and the bioinstructive role of surface topographies in vivo.

In summary, we show for the first time that unbiased screening of an algorithm generated topographical library in combination with machine learning algorithms can be used to identify topographies which promote both the attachment and polarization of macrophages in the absence of exogenous cytokines. Specifically, we found the importance of the pattern area of micropillars to be key in macrophage attachment and furthermore it is a combination of pattern area and density of these micropillars which modulate cell phenotype. As macrophages are key mediators of inflammatory and tissue repair processes, the ability of surface topography to mediate changes in cell phenotype provides a powerful tool in the goal of achieving rationale design of “immune‐instructive” biomaterials for implantable medical devices. Within the context of biomaterials discovery and immune‐bioengineering, this offers a defined platform and robust strategy not only for new and novel applications but also for understanding the basic biological mechanisms underlying these phenomena, although there is more work required to provide a mechanistic description of the observations discovered herein.

## Experimental Section

##### Monocyte Isolation

Anonymous buffy coat samples from healthy donors (aged 17–66) were obtained from the National Blood Service (National Blood Service, Sheffield, UK) after obtaining written informed consent and with local Ethics Committee approval (2009/D055). Peripheral blood mononuclear cells (PBMCs) were isolated from heparinized blood by Histopaque‐1077 (Sigma‐Aldrich) density gradient centrifugation. Monocytes were isolated from PBMCs using the MACS magnetic cell separation system (positive selection with CD14 MicroBeads and LS columns, Miltenyi Biotec) as described previously.^[^ ^20^, ^40^ ^]^

##### Cell Culture

Purified monocytes were suspended in RPMI‐1640 medium supplemented with 10% fetal bovine serum (FBS), 2 mm L‐glutamine, 100 U mL^−1^ penicillin, and 100 µg mL^−1^ streptomycin (all from Sigma‐Aldrich) (henceforth referred to as “complete medium”) and seeded at three million cells per well in 6‐well polystyrene plates (Corning Life Sciences).

##### TopoChip Fabrication

A detailed description of the surface fabrication procedures can be found elsewhere.^[^ ^28^, ^41^ ^]^ Briefly, the TopoChip was comprised of 2176 unique surface topography combinations along with flat planar controls, printed on a polystyrene (PS) chip. Topographies were generated based on a random in silico combination of primitive shapes (circles, triangles, rectangles). Each individual TopoUnit (dimensions: 300 × 300 µm) contains a different kind of topography (composed of different primitive shapes) all at a height of 10 µm. Each chip (dimensions: 2 × 2 cm^2^, 66 × 66 TopoUnits) contained internal duplicates for every TopoUnit. The location of each TopoUnit was the same on every TopoChip and to rule out location bias, duplicate arrays were placed diagonally to each other. TopoChips were made from polystyrene (PS) by hot embossing PS films (as described in Vermeulen et al.,).^[^ ^31^ ^]^ Prior to cell culture, TopoChips were treated with oxygen plasma for 30 s (Zepto low cost plasma cleaner, Diener electronic), placed in a 6‐well tissue culture‐treated polystyrene (TCP) plates (Corning Life Sciences), and incubated with complete media for 1 h at 37 °C.

##### Surface Chemistry Analysis

The surface chemistry of selected areas of the TopoChip was assessed using time‐of‐flight secondary ion mass spectrometry (ToF‐SIMS) and XPS.

##### ToF‐SIMS

Measurements were conducted using an IONTOF HybridSIMS (3D OrbiSIMS) instrument employing a 30 keV Bi3+ primary ion source. A 0.3 pA target current primary ion beam was rastered over a 150 × 150 µm area 256 × 256 pixels with both positive and negative secondary ions. Ion masses were determined using a high resolution Time‐of‐Flight analyser in delayed extraction mode and the ion dose was kept < 1 × 10^12^ ions cm^−2^ to ensure static conditions.

##### XPS analysis

Samples were analyzed using the Kratos AXIS ULTRA with a mono‐chromated Al k*α* X‐ray source (1486.6 eV) operated at 10 mA emission current and 12 kV anode potential (120 W) Spectra were acquired with the Kratos VISION II software. A charge neutralizer filament was used to prevent surface charging. Hybrid‐slot mode was used measuring a sample area of approximately 300 × 700 µm. The analysis chamber pressure was better than 5 × 10^−9^ mbar. Three areas per sample were analyzed. A wide scan at low resolution (Binding energy range 1400–−5 eV, with pass energy 80 eV, step 0.5 eV, sweep time 20 min) The wide scan spectra were used to estimate the total at.% of the detected elements. High resolution spectra at pass energy 20 eV, step of 0.1 eV, and sweep times of 10 min each were also acquired for photoelectron peaks from the detected elements and these were used to model the chemical composition. The spectra were charge corrected to the C1s peak (adventitious carbon or a known polymer CH2 or CH3 peak) set to 285 eV.

CasaXPS (version 2.3.18dev1.0x) software was used for quantification and spectral modeling. The measured N 1s fraction in medium conditioned surfaces was converted into protein layer thickness using Ray and Shard (2011) relationship between [N] and protein depth on the surface.

##### TopoChip Imaging and Data Acquisition

All samples were inverted, and fluorescent images acquired using a Zeiss Axio Observer Z1 microscope (Carl Zeiss, Germany) equipped with a Hamamatsu Flash 4.0 CMOS camera and a motorized stage for automated acquisition. A Zeiss, EC Plan‐Neofluar 20×/0.50 Ph 2) was used to provide sufficient resolution to capture the fluorescence data whilst enabling the use of the auto‐focus function, which considerably reduced scanning times and file sizes per TopoChip. Images were cropped to a smaller field of view (280 x 280 µm) that did not include the walls of the TopoUnits to improve the auto‐focus function. Following acquisition, images were manually inspected to identify out of focus images which were removed from the analysis. Subsequently, all individual TopoChip images were analyzed using open source software Cell Profiler (CP) using custom made pipelines. After illumination corrections, nuclei were detected as the primary objects using the Robust Background thresholding method applied globally on the DAPI channel. Subsequently, cell demarcation and morphology were determined by applying a Watershed gradient method with background thresholding applied and appropriate propagation algorithms on the CellMask (plasma membrane) channel. Cells found to be in contact with the edges were filtered out of the dataset. For cell phenotyping analysis, the mean fluorescent intensity value inside the segmented cell area is summarized to determine the value for each respective fluorescent channel.

##### Computational Analysis

To identify the surface design parameters that can influence monocyte adhesion, monocyte attachment screening data of CD14+ human monocytes on 30 s plasma treated polystyrene TopoChips were studied. Data was first pre‐processed and, for each donor, the values quantifying mean fluorescence of Calprotectin, MR, and the total cell count per topography were normalized by their corresponding flat topography values. As cell fluorescence and attachment may be heterogeneous due to poor representation on the slide, replicates by donor were averaged, and those TopoUnits with SNR lower than two were excluded from the analysis for most cases. There were circumstances of low attachment, however, where the SNR values were carefully moderated by the standard deviation values. Subsequently, average, standard deviation, and SNR were calculated between donors for the modeling studies.

As attachment and polarization were both important, machine learning models were trained to predict initially attachment; subsequently, a composite dependent variable Log(M2/M1) × Attachment to investigate attachment associated with polarization properties was investigated. The TopoUnit topographies are computationally described using a combination of surface feature parameter values used to construct the features in addition to Cell Profiler generated parameters (from bright field images) which describe characteristics of surface feature area and shape. A total of 246 descriptors was investigated. SHAP method was employed for feature selection to eliminate uninformative and less informative descriptors. SHAP was implemented using the shap package in Python 3.7.^[^ ^42^ ^]^ Regression models were generated using random forest and XGBoost, using the packages sklearn and xgboost in Python 3.7. 70% of the data instances were employed for model training and 30% for testing.^[^ ^43^, ^44^ ^]^ The performance of the predictive models and the topographical descriptors that contributed most strongly to the attachment and polarization were consistent for both methods. Results for XGBoost are shown in Figures S2 and S5. The figure presents the results of the regression models as well as the features selected. The features are ordered from top to bottom based on their average impact on the model output magnitude.

##### Feature Descriptor Generation

In addition to topography design descriptors that were extracted directly from the design file and were reported elsewhere, an additional set of features were obtained as following: topographies designs were represented as black and white (binary) images where white corresponded to the design of the pillars and black to the spacing between them.^[^ ^28^ ^]^ Images were created from the design file of the topographies in custom Matlab 2017 script. Only images of unique topographical features and spacing around them (Feature Block) were used. Ten pixels on the resulted images corresponded to the 1 µm on real fabricated surfaces. Shape and Size related Surface Descriptors were extracted via custom build image analysis pipeline constructed in CellProfiler 2.2.

For quantification of the spacing between pillars Feature Block binary images were inverted and replicated across the area that corresponds to real fabricated surfaces. To reduce the size of the resulted images they were downscaled five times. We further employed MaxInscribed Circles algorithm as described here https://imagej.net/Max_Inscribed_Circles. To identify the size and number of circles that can be fitted in the gap between pillars. The algorithm is looped until a circle diameter smaller than three pixels is found.

Further analysis was performed in R version 3.5 unless specified differently. Per topography, summary statistics of topography design descriptors such as mean, median, percentile, number of pillars, was quantified. This generated a library of 246 topographical descriptors, subsequently, Pearson correlation analysis was applied to remove overlapping and non‐intuitive descriptors (≥0.85). This finalized descriptor library of 65 physical surface determinants was used for modeling and correlative analysis.

##### Immunocytochemistry—For Attachment Experiments

Cells were fixed with 4% paraformaldehyde (Bio‐Rad) in PBS as described above, washed thrice with PBS (5 min per wash), then permeabilized by 0.2% Triton‐X100 (Sigma‐Aldrich) in PBS for 20 min. After three washes with PBS, non‐specific binding was blocked with 5% goat serum in PBS as described in the previous section. This was followed by two washes with PBS and incubation with the cellular stain, CellMask (Invitrogen) in PBS for 30 min. Cells were then washed three times with PBS and stained with 250 ng mL^−1^ DAPI (4′,6‐Diamidino‐2‐Phenylindole) (Invitrogen) in PBS for 5 min, washed three times with PBS, then mounted with anti‐fade medium (Pro‐Long Gold), and on a standard microscope slide followed by imaging using a fluorescent microscope (Zeiss).

##### Immunocytochemistry—For Phenotypic Analysis

Adherent cells on coverslips were fixed with 4% paraformaldehyde (Bio‐Rad) in PBS for 10 min. Fixation and all subsequent steps in this procedure were carried out at room temperature; all washes were carried out with 0.2% Tween 10 (Sigma‐Aldrich) in PBS (5 min per wash) except where stated. Following fixation, cells were washed three times, then blocked with 1% (w/v) glycine (Fisher Scientific) and 3% (v/v) bovine serum albumin (BSA, Sigma‐Aldrich) in PBS for 30 min. Subsequently, cells were washed twice and incubated for 30 min with 5% (v/v) goat serum (Sigma‐Aldrich) in PBS to block non‐specific antibody binding. Next, cells were incubated for 1 h with the appropriate primary antibody (see Table 1, Supporting Information), washed three times, and then incubated for 1 h with the appropriate secondary antibody at room temperature (see Table 1, Supporting Information). For intracellular staining experiments, cells were treated with Brefeldin‐A (1:1000) (Thermo Fisher) for 16 h prior to fixation before processing as described above. Cells were stained with 2 µg mL^−1^ anti‐human TNF*α* (IgG1) mAb (Abcam), and with 1 µg mL^−1^ anti‐human IL‐10 (IgG1) mAb (Abcam) followed by 1 h incubation at room temperature. After washing, cells were stained with 8 µg mL^−1^ Rhodamine‐x goat anti‐mouse IgG (H + L) secondary Ab (Invitrogen), and 8 µg mL^−1^ Alexa flour‐647 goat anti‐rabbit IgG (H + L) secondary antibody (Invitrogen) for another hour at room temperature. Finally, in all experiments, cells were stained with 250 ng mL^−1^ DAPI (4′,6‐Diamidino‐2‐Phenylindole) (Life Technologies) in PBS for 5 min, washed three times with PBS, then mounted with anti‐fade medium (Pro‐Long Gold), on a standard microscope slide followed by imaging using an automated fluorescent microscope (Zeiss).

##### Cytokine Polarization

For cytokine polarized controls, monocytes were isolated as described previously and plated onto tissue culture plastic and subsequently exposed to various conditions to drive a desired polarization state. Polarized phenotypes were generated following addition of cytokines (final concentrations); M0—M‐CSF (10 ng mL^−1^), M1—GM‐CSF (20 ng mL^−1^), and IFN‐*γ* (20 ng mL^−1^) or M2; M‐CSF (50 ng mL^−1^) & IL‐4 (20 ng mL^−1^). On day 6, cells were fixed and stained for immunofluorescent staining as described above.

##### ECM Binding Assay

Prior to cell seeding, TopoChips were incubated with either human fibronectin or rat tail collagen I at a final concentration of 10 µg mL^−1^ (both Sigma‐Aldrich). To coat TopoChips, stock solutions were diluted in sterile PBS and coated with minimal volume for 3 h at room temperature. TopoChips were then air dried for 45 min at room temp before UV sterilization and cell seeding as described earlier.

##### In Vivo* *Murine Model

Sections of polyurethane TopoChip surfaces (0.3 × 5 mm) were prepared and served as a model implant. Sterilization consisted of exposure to ultraviolet light for a period of 20 min. All in vivo studies were approved by the University of Nottingham Animal Welfare and Ethical Review Board and were carried out in accordance with home office authorization under project license number 30/3238. Age‐matched adult female BALB/C mice, Charles River, were housed in IVC under 12 h light cycle with food and water ad libitum. An hour before catheter implantation, analgesia (carprofen) was administered subcutaneously (2.5 mg per kg), animals where anesthetized and hair removed by shaving, the area was sterilized with Hydrex (Ecoblab). A small incision was made in the flank and individual TopoChip samples were loaded into a trocar needle (9 g) and injected subcutaneously on one side of the mouse. The wound was sealed using Gluture skin glue. All mice were monitored until they recovered from the anesthesia and inflammation at the site of implantation, behavioral changes and other adverse reactions were monitored throughout the duration of the experiment. At the end of the experiment, on day 10, mice were humanely sacrificed by CO_2 _euthanasia. Subsequently, samples were excised and fixed in formal saline overnight, washed in PBS and permeabilized with 100% ethanol. Following a further wash in PBS, samples were stained with 4′,6‐diamidino‐2‐phenylindol (DAPI) for 30 min followed by FM1‐43 dye for 3 min. Finally, samples were washed (PBS) and mounted on a coverslip using sigma fluoromount for imaging.

## Conflict of Interest

The authors declare no conflict of interest.

## Supporting information

Supporting Information
