# Supplementary material for: Immune Modulation by Design: Using Topography to Control Human Monocyte Attachment and Macrophage Differentiation
Source: Adv Sci (Weinh). 2020 Apr 28;7(11):1903392. doi: 10.1002/advs.201903392 (PMC7284204; doi:10.1002/advs.201903392)
Supplement: Supplementary file 1 — Supporting Information [file ADVS-7-1903392-s001.docx]

# Supporting Information

Title: Immune modulation by design: using topography to control human monocyte attachment and macrophage differentiation

Author(s), and Corresponding Author(s)* ((write out full first and last names))

Matthew J Vassey, Grazziela P Figueredo, David Scurr, Aliaksei S Vasilevich, Steven Vermeulen, Aurélie Carlier, Nick Beijers, Dave Winkler, Jan de Boer, Amir Ghaemmaghami* and Morgan R Alexander*

*All relevant data are available from the University of Nottingham’s Research Data Management Repository* [*https://rdmc.nottingham.ac.uk*](https://rdmc.nottingham.ac.uk) *(DOI: 10.17639/nott.7050)”*


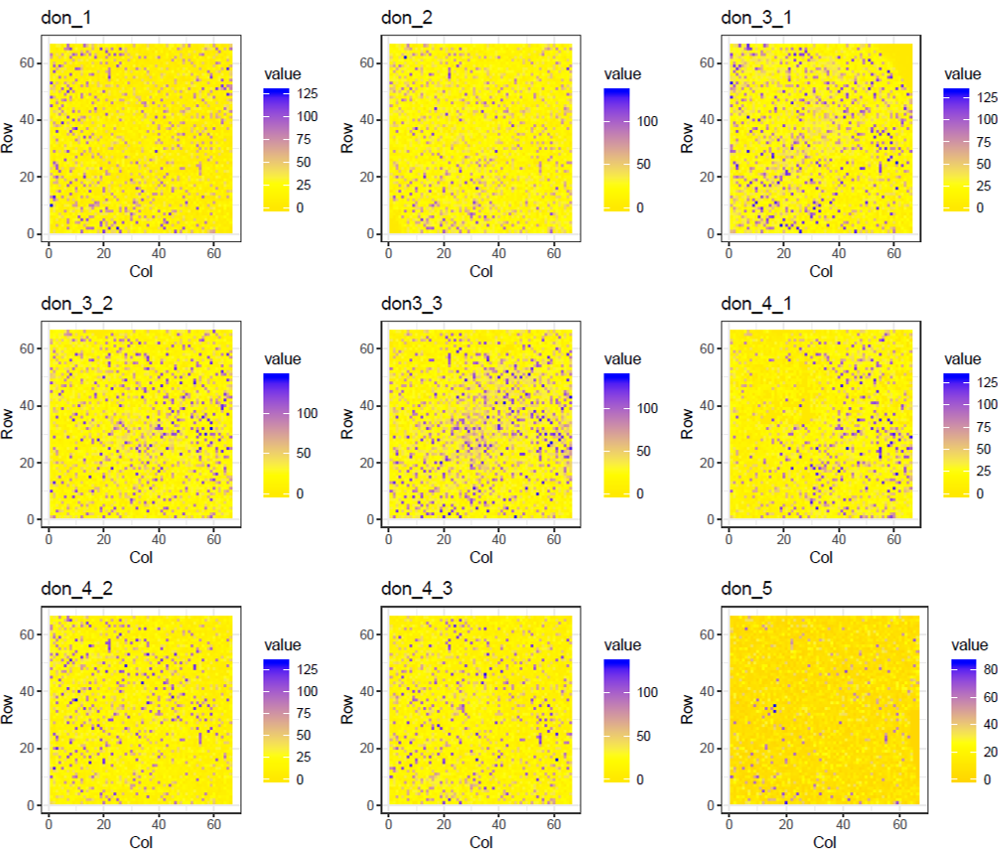


Figure S1 – Heat map analysis of cell attachment (per donor)


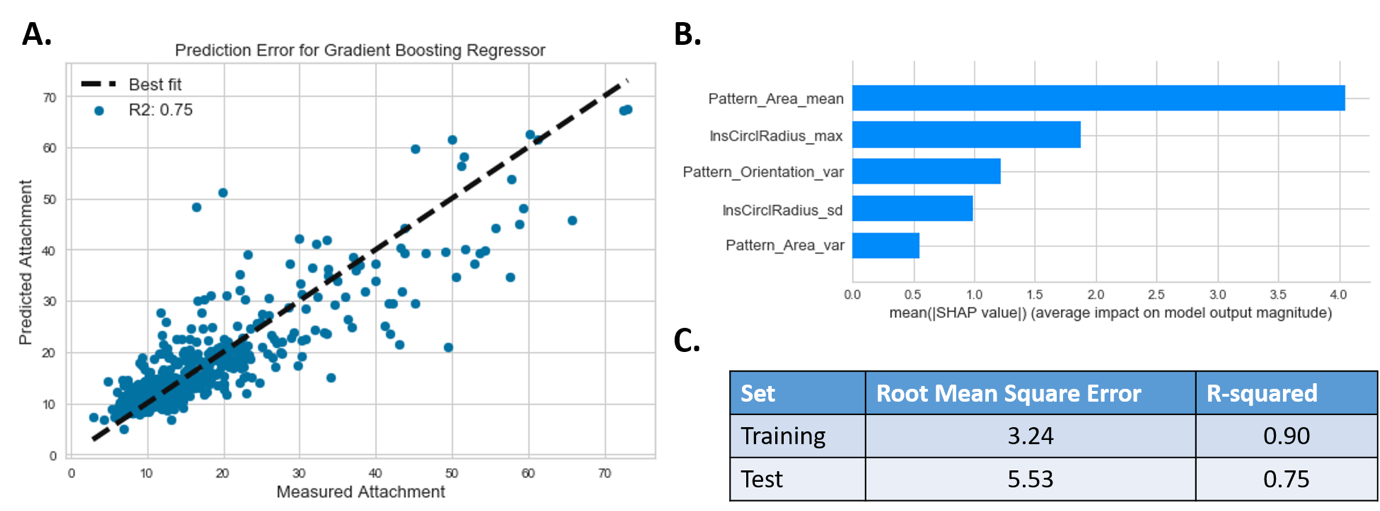


Figure S2 – Machine Learning modelling results for macrophage attachment using XGBoost (a) Scatter plot of the measured against predicted values (b) SHapley Additive exPlanation (SHAP) analysis of the surface structure descriptors ranked by their average impact on model output and (c) table of results containing the random mean square error (RMSE) and R^2^ values for the prediction model for training and test sets.


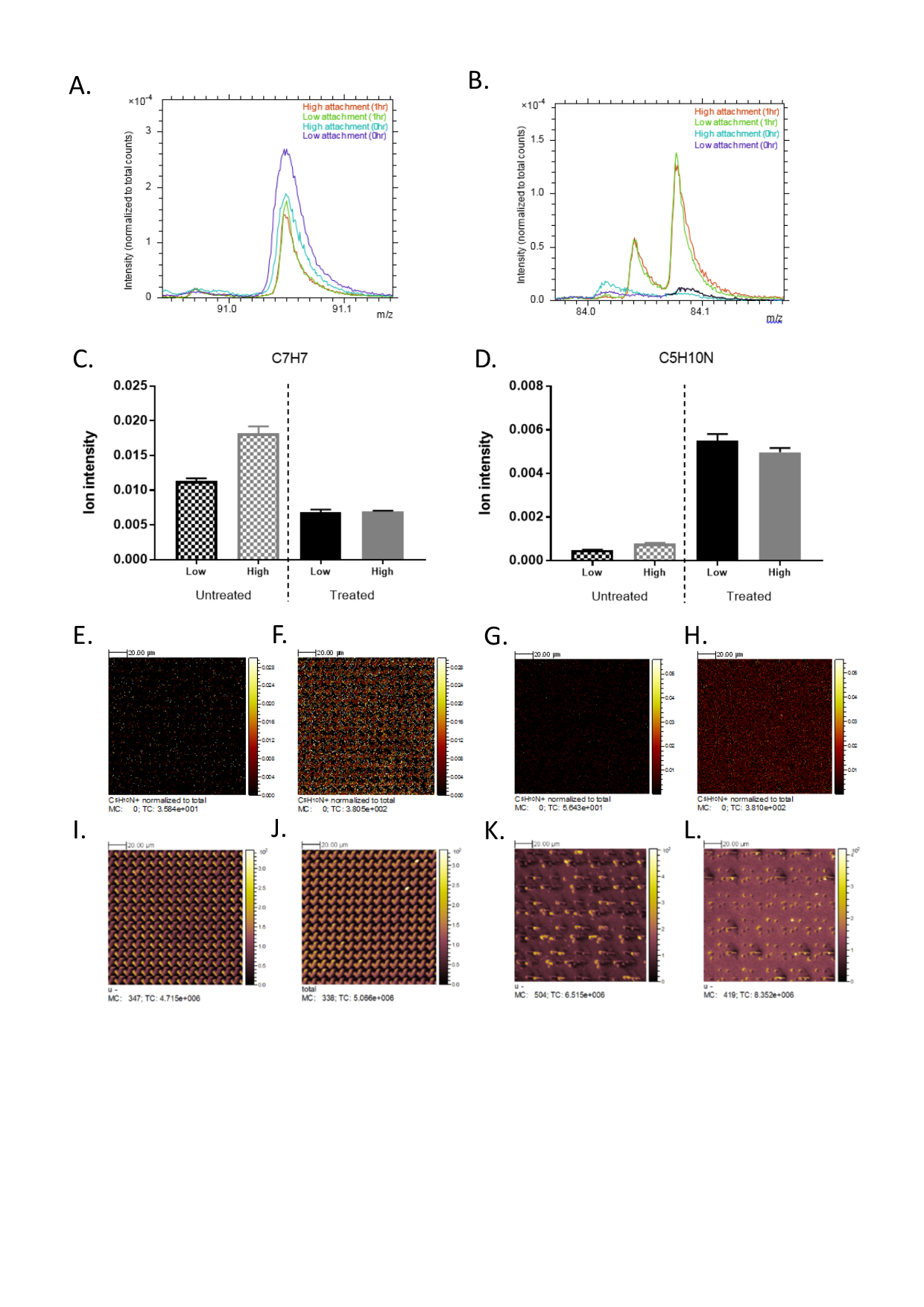


Figure S3 –Surface characterisation of TopoChip surface chemistry by 3D MS imaging and SIMS analysis. TopoChips were incubated with or without RPMI Complete Media (see SI materials and methods) for 1hour. Comparison of peak ion intensities of the TopoChip surfaces of high (A) and low (B) macrophage attachment. Mass peaks m/z 91 (C7H7+) and 84 (C5H10N+) were used to identify the base substrate (C) and lysine as a protein marker (D), respectively.


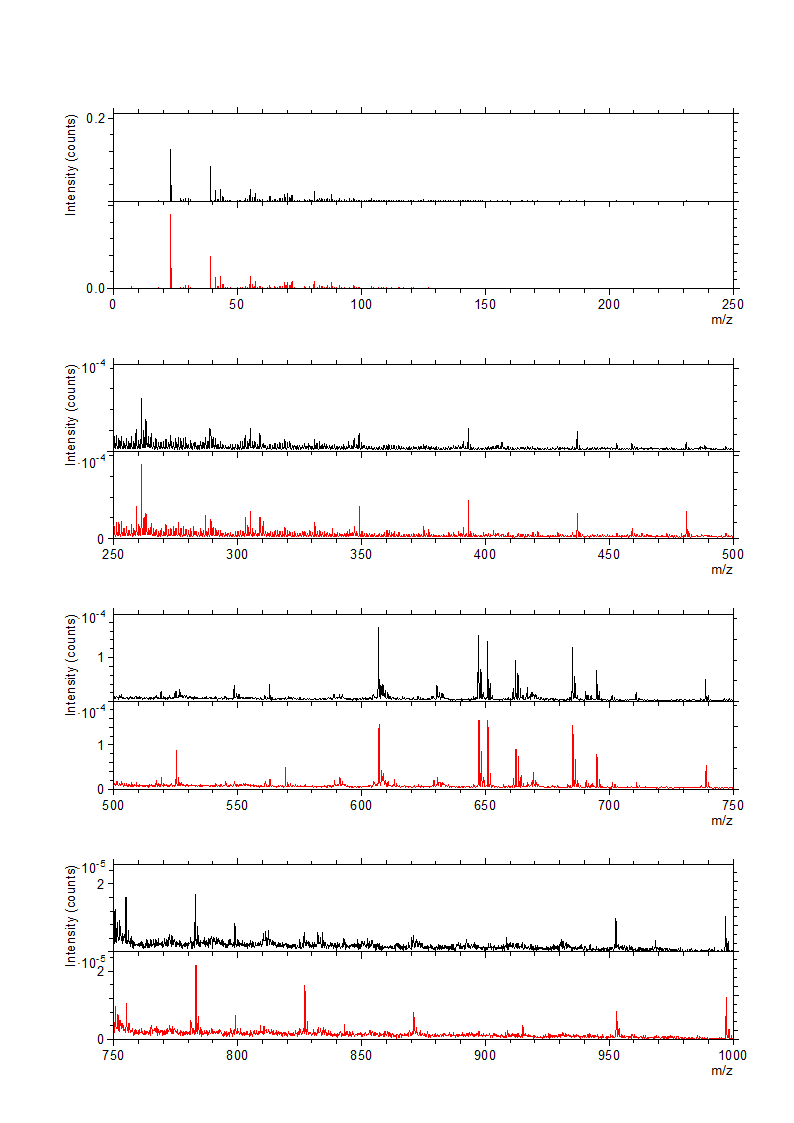


Figure S4 – ToF SIMS positive polarity spectra acquired for media exposed TopoUnits compared for high (Black) and low (Red) attachment (m/z 0-1000).


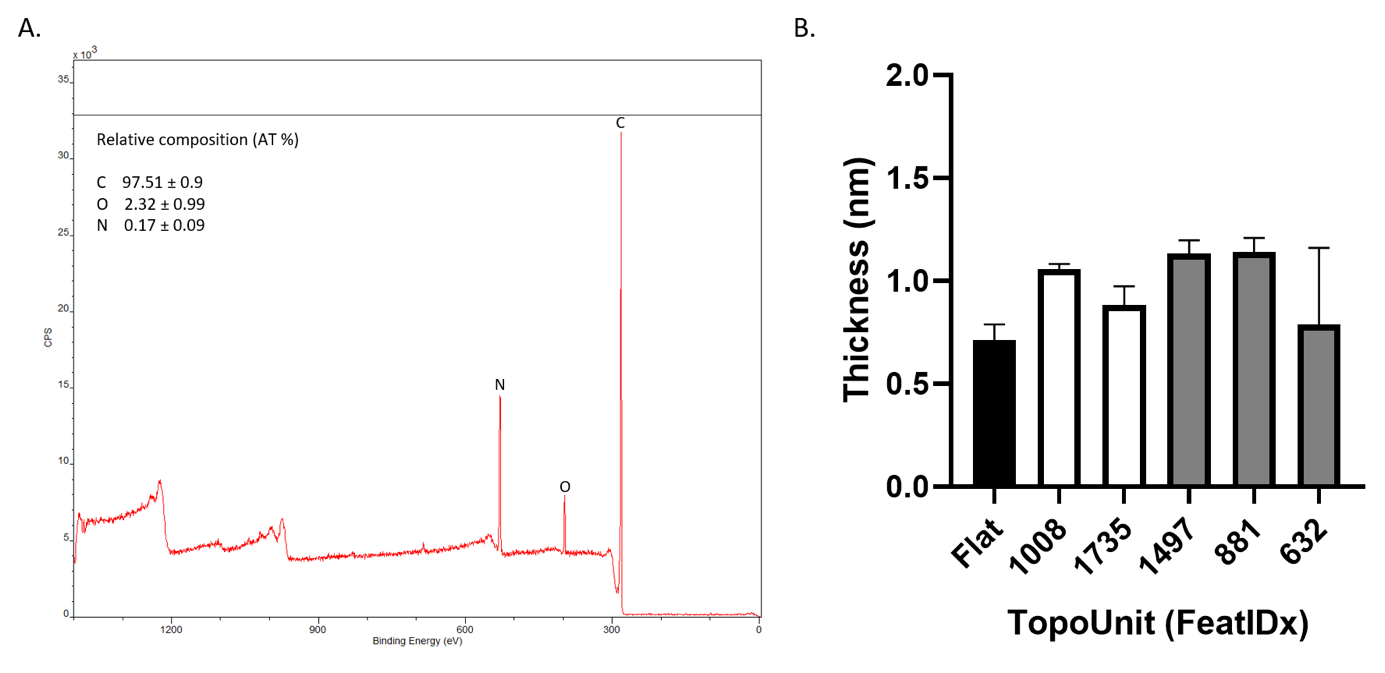


Figure S5 – XPS elemental analysis of TopoUnit surfaces. (a) Representative XPS spectrum, obtained from a 100 × 100 μm area corresponding to a flat planar plasma-treated surface. Elemental composition for C, O and N elements are shown (atomic %). (b) Protein layer thickness on a range of high (white bars) and low attachment (grey bars) TopoUnit surfaces compared to the flat area (black bar). Data expressed as protein layer thickness +/- standard deviation of at least three independent samples.


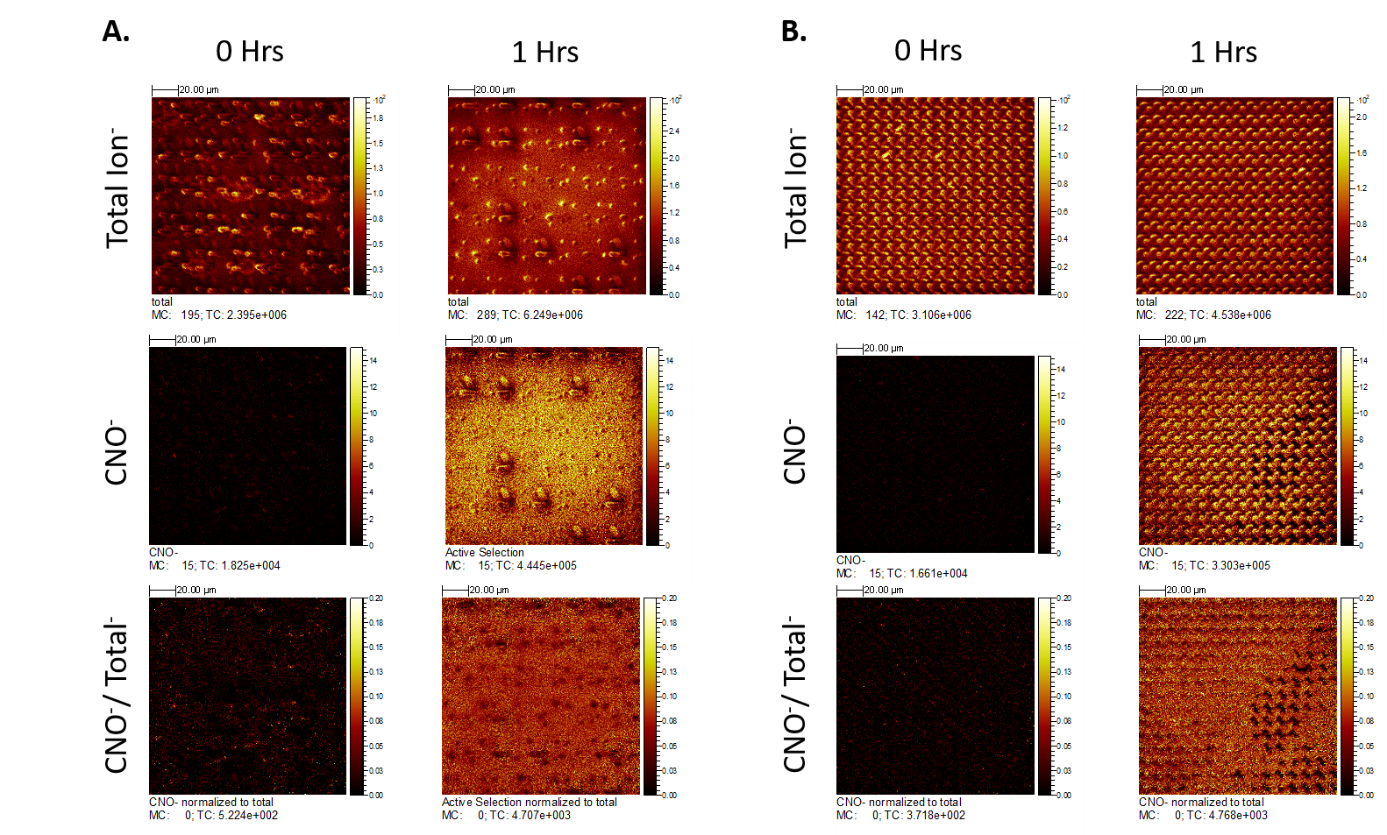
Figure S6 - ToF MS images of relative total ion distribution, CNO^-^ and CNO- normalised to total ion count; images show regions of untreated and 1 hr treated on (A) high and (B) low attachment TopoUnits, respectively. Images representative of three independent areas analysed per sample. Data acquired with 30 keV Bi3+ (lateral resolution ~2 μm, pixel size 2 μm)


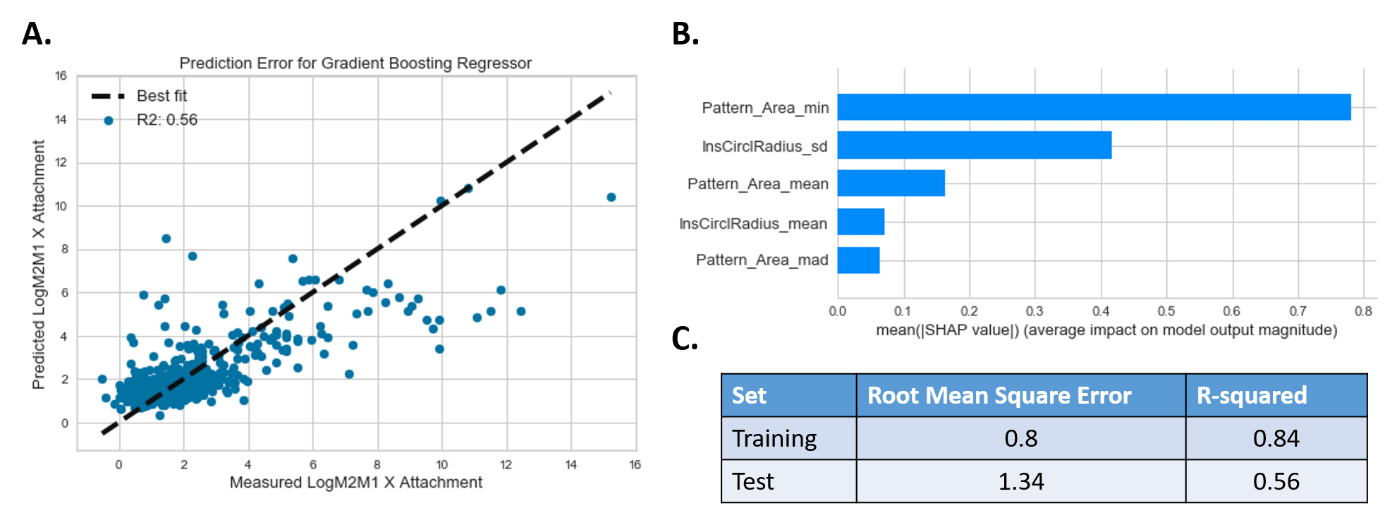


Figure S7 - Machine Learning modelling results for macrophage polarisation using XGBoost (a) Scatter plot of the measured against predicted values (b) SHapley Additive exPlanation (SHAP) analysis of the surface structure descriptors ranked by their average impact on model output and (c) table of results containing the random mean square error (RMSE) and R^2^ values for the prediction model for training and test sets.


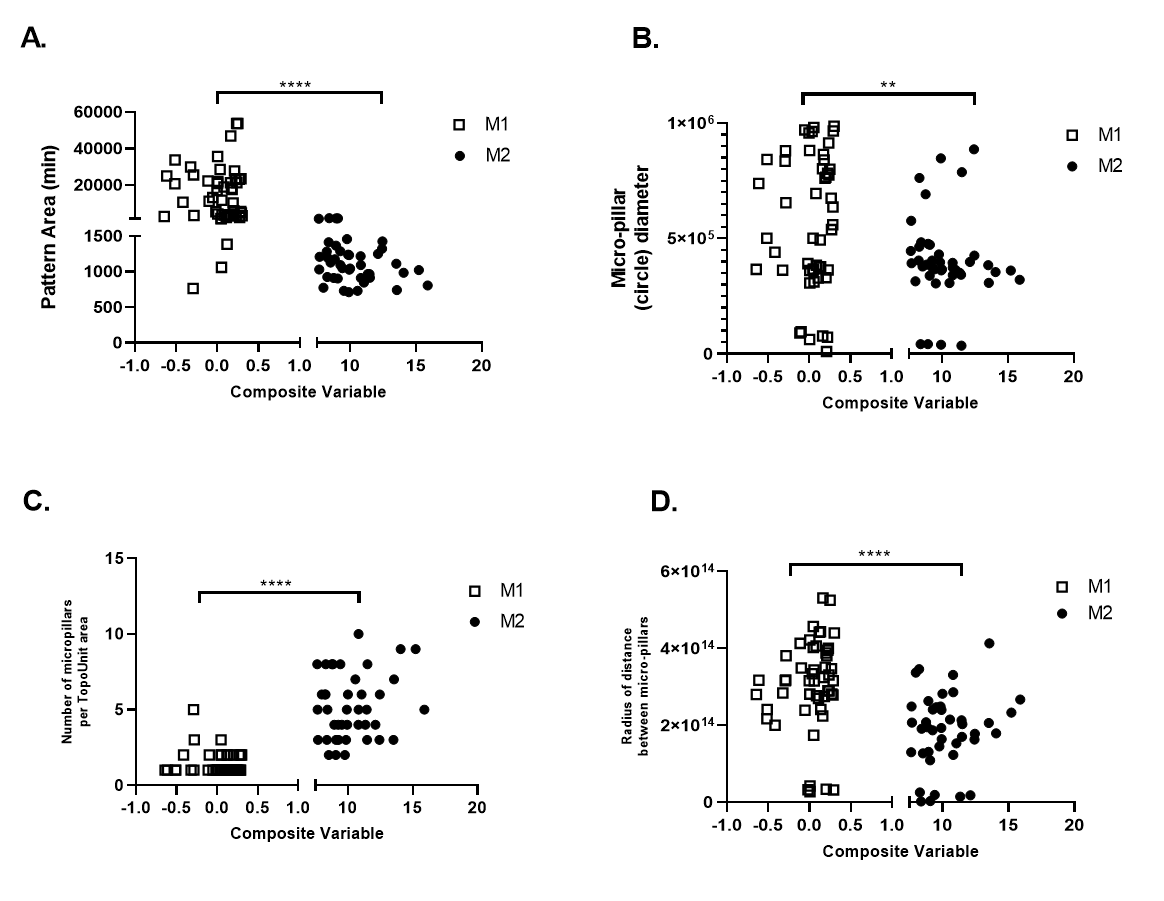


Figure S8 – TopoUnit descriptors of important surface features compared to the composite variable “Log(M2/M1) x attachment” to represent phenotype importance in high attachment (□ and • represent the top 50 M1 and M2 performing TopoUnits, respectively). Statistical significance of P < 0.05 determined using Mann-Whitney test.

Supplementary Table 1: TopoUnit topography surface descriptors.

| **Surface property** | **Description** |
| --- | --- |
| NumTri | The number of triangles used |
| NumLine | The number of lines used |
| CircDiam | Circle diameter |
| TriSize | Length of the shortest side of a triangle |
| LineLen | Line length |
| RotSD | The standard deviation (in degrees), is used to determine the rotation of the primitives when they are placed in the feature |
| CircArea | The area of circle primitives |
| TriArea | The area of triangle primitives |
| LineArea | The area of line primitives |
| DT | The number of triangle primitives scaled by feature area |
| DL | The number of line primitives scaled by feature area |
| CA | The total area of circle primitives scaled by feature area |
| TA | The total area of triangle primitives scaled by feature area |
| CCD | Number of colour changes of the feature over the diagonal |
| Pattern Count | Number of micro-pillars per TopoUnit area |
| Area^2^ | The actual number of pixels in the region |
| Compactness^2^ | The variance of the radial distance of the object's pixels from the centroid divided by the area |
| Eccentricity^2^ | The eccentricity of the ellipse that has the same second-moments as the region. The eccentricity is the ratio of the distance between the foci of the ellipse and its major axis length. The value is between 0 and 1. (0 and 1 are degenerate cases; an ellipse whose eccentricity is 0 is actually a circle, while an ellipse whose eccentricity is 1 is a line segment.) |
| Extent^2^ | The proportion of the pixels in the bounding box that are also in the region. Computed as the Area divided by the area of the bounding box. |
| Form Factor^2^ | Calculated as 4*π*Area/Perimeter2. Equals 1 for a perfectly circular object. |
| Major axis length^2^ | The length (in pixels) of the major axis of the ellipse that has the same normalized second central moments as the region. |
| Min Feret Diameter^2^ | The Feret diameter is the distance between two parallel lines tangent on either side of the object (imagine taking a caliper and measuring the object at various angles). The minimum Feret diameter is the smallest possible diameter, rotating the calipers along all possible angles. |
| Median Radius^2^ | The median distance of any pixel in the object to the closest pixel outside of the object. |
| Max Radius^2^ | The maximum distance of any pixel in the object to the closest pixel outside of the object. For skinny objects, this is 1/2 of the maximum width of the object. |
| Orientation^2^ | The angle (in degrees ranging from -90 to 90 degrees) between the x-axis and the major axis of the ellipse that has the same second-moments as the region. |
| Perimeter^2^ | The total number of pixels around the boundary of each region in the image. |
| Solidity^2^ | The proportion of the pixels in the convex hull that are also in the object, i.e. ObjectArea/ConvexHullArea. Equals 1 for a solid object (i.e., one with no holes or has a concave boundary), or <1 for an object with holes or possessing a convex/irregular boundary. |
| Inscribed Circle number^2^ | The number of inscribed circles of a defined minimum diameter found between objects |

Each micro-topographical element contains primitives (circles, triangles and rectangles). Features are repeated to cover the surface of a TopoUnit. ^2^For each of the descriptors derived from Image Analysis of bright field images, area and shape features are extracted, each parameter has an additional subset of descriptors including; standard deviation, mean, median, mad, minimum, maximum, variance, skewness, mode and percentile (0.1, 0.25, 0.5, 0.75 and 0.9) measurements (feature descriptions adapted from CellProfiler.org).
